# Engineering *Escherichia coli* for autoinducible production of L-valine: An example of an artificial positive feedback loop in amino acid biosynthesis

**DOI:** 10.1371/journal.pone.0215777

**Published:** 2019-04-25

**Authors:** Natalia V. Geraskina, Elena V. Sycheva, Valery V. Samsonov, Natalia S. Eremina, Christine D. Hook, Vsevolod A. Serebrianyi, Nataliya V. Stoynova

**Affiliations:** Ajinomoto-Genetika Research Institute, Moscow, Russian Federation; Nanjing University of Science and Technology, CHINA

## Abstract

Artificial metabolically regulated inducible expression systems are often used for the production of essential compounds. In most cases, the application of such systems enables regulating the expression of an entire group of genes in response to any internal signal such as an aerobic/anaerobic switch, a transition to stationary phase, or the exhausting of essential compounds. In this work, we demonstrate an example of another type of artificial autoinducible module, denoted a positive feedback module. This positive feedback module generates an inducer molecule that in turn enhances its own synthesis, promoting an activation signal. Due to the use of acetolactate, an intermediate of the L-valine biosynthetic pathway, as a specific inducer molecule, we realized a positive feedback loop in the biosynthetic pathway of branched chain amino acids. Such positive feedback was demonstrated to improve the production of a target compound.

## Introduction

At the present time, many useful substances, e.g., L-amino acids, biofuels, and fine chemicals, are produced at a large scale by microbiological fermentation [1–5]. For this purpose, specific strain-producers are required. The basic approach for developing a producing strain is activating a biosynthetic pathway making the desired substance by enhancing key enzyme gene expression. For this goal, many specific regulatory elements, native or artificial, and metabolic toggle switches are widely used for metabolic flux redirection [6–9]. For example, the gene of interest may be placed under control of a well-characterized regulatory region, such as the *lac* promoter, *trp* promoter, P_R_ or P_L_ promoters of λ phage, and *tac* promoter [10]. These promoters have different strengths and, in the absence of corresponding repressors, provide high constitutive expression of the target genes. There are also inducer-free expression systems based on growth phase- or stress-specific promoters, such as the promoter of the *rpsF* operon or the promoter of the *pst* operon in gram-positive bacteria [11, 12].

To construct novel genetic circuits, accurate predictive design of regulatory region from versatile components can be used to “reprogram” the behavior of living microorganisms [6, 13, 14]. At the same time, exploiting dynamic sensor-regulator systems (DSRSs) to achieve the desired expression level of target genes has become increasingly popular in metabolic engineering. The DSRSs use a transcription factor that senses a key intermediate and dynamically regulates the expression of genes involved in the target compound biosynthesis in response to intracellular metabolic states [15, 16].

In the present study, an artificial autoinducible expression module with positive feedback for enhancing target gene(s) expression was constructed on the basis of one of the known DSRSs from the metabolic pathway for L-valine and L-isoleucine biosynthesis in *Escherichia coli*. L-valine, an essential nutrient for animals and humans, can be produced by microbial cultivation technology together with metabolic engineering [2, 17–20]. The development of bacterial strains with higher production of L-valine is therefore of considerable interest.

Traditionally, expression systems can be induced in response to any internal signal such as an aerobic/anaerobic switch, a transition to stationary phase, or the exhaustion of essential compounds, which were used as autoinducible signals. In this work, we demonstrate an example of another type of artificial autoinducible expression element, the positive feedback module (PFM). The PFM generates an inducer molecule that in turn enhances its own synthesis, promoting an activation signal.

Our system is based on an endogenous LysR-type protein-regulated expression module of bacteria. Despite the great interest for exploiting autoinducible gene expression systems in metabolic engineering, we found no data describing the use of LysR-type proteins in artificial expression modules working in such a mode. Originally, LTTRs (LysR-type transcriptional regulators) were described as transcriptional activators of a single divergently transcribed gene, exhibiting negative autoregulation [21–23]. Extensive research has now led to them being regarded as global transcriptional regulators, acting as either activators or repressors of single or operonic genes; they are often divergently transcribed but can be located elsewhere on the bacterial chromosome [24]. Regulation is mediated by a co-inducer of LTTR proteins. A precursor for a useful metabolite or a substrate for an enzyme under regulation may act in that capacity. A complex of a transcription regulator and its co-inducer may bind to the -35 promoter region and thus change the ability of RNA polymerase to initiate transcription of the regulated gene. Many LTTR-family members have been described in *E*. *coli* [22, 25]. They regulate the transcription of genes responsible for nitrogen source utilization, amino acid biosynthesis and catabolism, oxidative stress response and the detoxification of the cell. The following are several such examples: (*i*) CysB activates transcription of the genes involved in sulfur utilization and sulfonate-sulfur metabolism and acts in a complex with O-acetylserine [26]; (*ii*) MetR complexed with L-homocysteine controls transcription of several genes involved in methionine biosynthesis [27] and a gene involved in protection against nitric oxide [28]; (*iii*) ArgR in a complex with L-arginine represses transcription of its own synthesis and several genes involved in the biosynthesis and transport of arginine and the transport of histidine [29] and activates the transcription of genes responsible for arginine catabolism [30].

**Fig 1:**
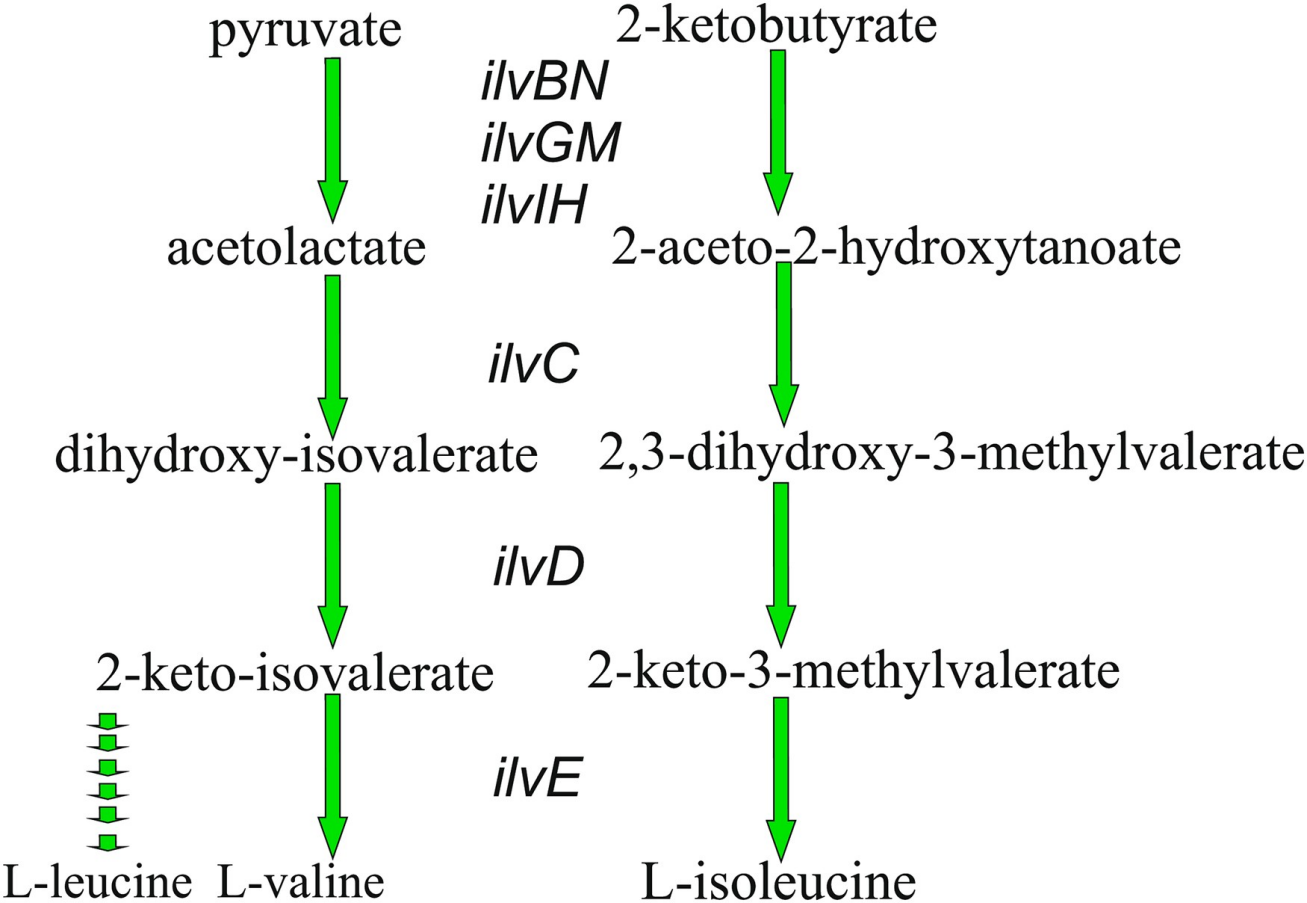
Schematics of BCAA biosynthesis. Metabolic pathway for BCAA (L-valine, L-leucine and L-isoleucine) synthesis consists of several reactions catalyzed by the following enzymes: acetohydroxy acid synthase I (encoded by *ilvBN* genes)/II (encoded by *ilvGM* genes)/III (encoded by *ilvIH* genes), isomeroreductase (encoded by the *ilvC* gene), dihydroxyacid dehydratase (encoded by the *ilvD* gene), and aminotransferase B (encoded by the *ilvE* gene).

**Fig 2:**
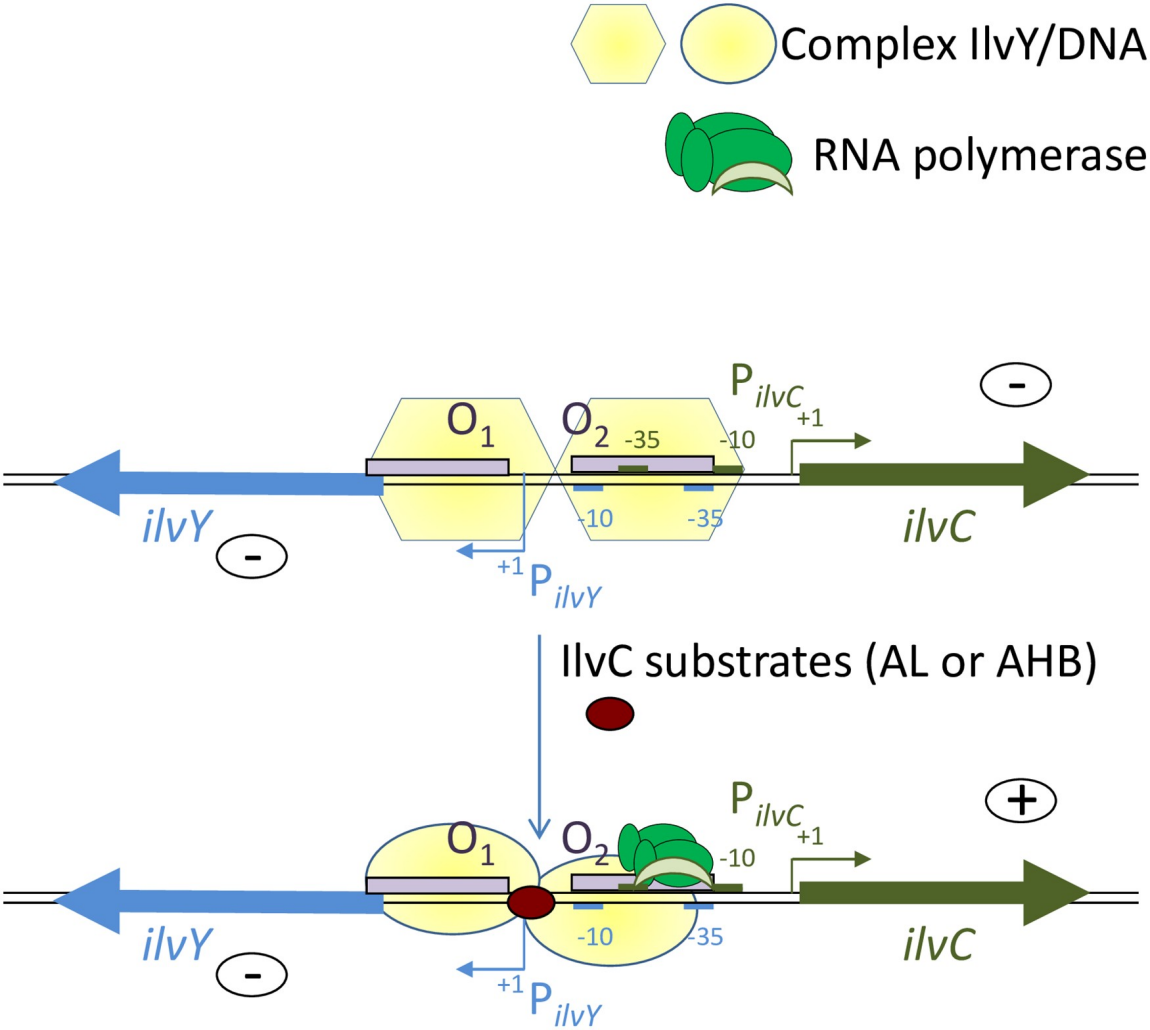
Schematic of the transcription regulation of the *ilvY* and *ilvC* genes. IlvY is the LysR-type transcriptional regulator designated in yellow. IlvY binds in a highly cooperative fashion to two tandem operator regions, O1 and O2, in the divergently overlapping *ilvYC* regulatory region designated $\left(\text{O}1/{\overset{⃐}{\text{P}}}_{ilvY},{\overset{⃑}{\text{P}}}_{ilvC}\text{/O}2\right)$. Upon binding to the first operator region, O1, the IlvY regulator negatively autoregulates transcription from the *ilvY* promoter, thus repressing its own further synthesis. Activation of *ilvC* transcription requires binding of the IlvY regulator to the second operator region, O2, and additional binding of a co-inducer such as 2-acetolactate (AL) or 2-aceto-2-hydroxybutanoate (AHB) to a preformed IlvY/O2 complex. When a co-inducer (red oval) is bound, a conformational change in the protein/DNA complex remodels the -35 region of the *ilvC* promoter and drastically increases the RNA polymerase binding capacity (Rhee et al., 1998). P_*ilvC*_ indicates the promoter of the *ilvC* gene. P_*ilvY*_ indicates the promoter of the *ilvY* gene. The minus sign (-) indicates a negative influence on gene transcription, and the plus sign (+) indicates a positive influence on gene transcription.

In the present study, as a proof of concept using PFM, we utilized an expression module regulated by LysR-type protein to improve the production of L-valine. The metabolic pathway for L-valine (and L-isoleucine) synthesis comprises several reactions catalyzed by the following enzymes: acetohydroxy acid synthase I (AHAS I) (IlvBN)/AHAS II (IlvGM)/AHAS III (IlvIH), isomeroreductase (IlvC), dihydroxyacid dehydratase (IlvD), and aminotransferase B (IlvE) (Fig 1). IlvY-mediated inducible expression of the *ilvC* gene is well characterized [31–34]. The *ilvY* and *ilvC* genes are structurally coupled in the *E*. *coli* chromosome and transcribed from divergently arranged promoters that partially overlap in their “upstream” regions (Fig 2). Furthermore, 2-acetolactate (AL) and 2-aceto-2-hydroxybutanoate (AHB), substrates for IlvC, are co-inducers of transcription activator IlvY, which enhances *ilvC* gene expression (Fig 2). Thus, IlvC synthesis is activated in the presence of its own substrates.

We propose to enhance and maintain stable *ilvBN* gene expression by placing it under the control of the region regulating the *ilvC* gene. The product of the AHAS I-mediated reaction is AL or AHB (co-inducers of IlvY); thus, oversynthesis of these molecules leads to the activation of transcription from P_*ilvC*_ and should then increase AHAS I synthesis itself. Such autoinducible positive feedback regulation can supply an appropriate level of AHAS I and, consequently, AL, the precursor of the target product L-valine. The IlvY\P_*ilvC*_-determined autoinducible regulatory module shows the possibility to exploit such artificial positive feedback circuits, and their introduction into metabolic pathways for the development of industrial strains was demonstrated for the first time.

## Materials and methods

### Bacterial strains and growth conditions

The strains used in this study are shown in Table 1. The following media were used for bacteria cultivation: lysogeny broth (LB) [35]; minimal medium (M9), containing 11 g/L M9 minimal salts (Sigma, St. Louis, Missouri, USA), 4 g/L glucose, 10 mg/L CaCl_2_, 0.2 mg/L MgSO_4_; fermentation medium (FM), containing 60 g/L glucose, 15 g/L (NH_4_)_2_SO_4_, 1.5 g/L KH_2_PO_4_, 1 g/L MgSO_4_ × 7H_2_O, 0.1 g/L thiamine-HCl, 25 g/L CaCO_3_, with the addition of 10% (v/v) LB medium. The minimal medium for the strains that possessed an *ilvYC* deletion and AHAS-deficient strains was additionally supplemented with Ile (25 mg/L) and Val (25 mg/L). Ampicillin (Ap, 100 mg/L), kanamycin (Km, 50 mg/L) and chloramphenicol (Cm, 40 mg/L) were used for selection as necessary.

**Table 1:** Bacterial strains used in this study.

Strain	Description	Source
MG1655	*Escherichia coli*, K-12 wild-type	VKPM^a^ B6195
K12	*Escherichia coli*, K-12 wild-type	VKPM^a^ B7
K12 *cat*-*ilvY*-P_*ilvC*_-*lacZ*	K12 with expression unit *cat*-*ilvY*-P_*ilvC*_ upstream of *lacZ* gene	This work
K12 *cat*-P_tac_-*lacZ*	K12 with constitutive promoter P_tac_ upstream of *lacZ* gene	[36]
K12 2Δ	K12 derivative with deletions of *ilvGM* and *ilvIH* genes	This work
K12 3 Δ	K12 derivative with deletions of *ilvBN*, *ilvGM* and *ilvIH* genes	This work
K12 *cat*-*ilvY*-P_*ilvC*_-*lacZ* 3Δ	K12 3 Δ with expression unit *cat*-*ilvY*-P_*ilvC*_ upstream of *lacZ* gene	This work
K12 *cat*-P_tac_-*lacZ* 3Δ	K12 3 Δ with constitutive promoter P_tac_ upstream of *lacZ* gene	This work
K12 2Δ P_L_-*ilvBN*	K12 derivative with deletions of *ilvGM* and *ilvIH* genes, overexpression of *ilvBN* genes under control of “strong” constitutive promoter P_L_	This work
K12 *cat*-*ilvY*-P_*ilvC*_-*lacZ* 2Δ P_L_-*ilvBN*	K12 2Δ P_L_-*ilvBN* with expression unit *cat*-*ilvY*-P_*ilvC*_ upstream of *lacZ* gene	This work
K12 *cat*-P_tac_-*lacZ* 2ΔP_L_-*ilvBN*	K12 2Δ P_L_-*ilvBN* with constitutive promoter P_tac_ upstream of *lacZ* gene	This work
K12 2Δ P_L_-*ilvBN*^*fbr*^	K12 derivative with deletions of *ilvGM* and *ilvIH* genes, overexpression of *ilvBN*^*fbr*^ genes encoding feedback-resistant AHAS I under control of “strong” constitutive promoter P_L_	This work
K12 *cat*-*ilvY*-P_*ilvC*_-*lacZ* 2Δ P_L_-*ilvBN*^*fbr*^	K12 2Δ P_L_-*ilvBN*^*fbr*^ derivative with expression unit *cat*-*ilvY*-P_*ilvC*_ upstream of *lacZ* gene	This work
K12 *cat*-P_tac_-*lacZ* 2ΔP_L_-*ilvBN*^*fbr*^	K12 2Δ P_L_-*ilvBN*^*fbr*^ derivative with constitutive promoter P_tac_ upstream of *lacZ* gene	This work
K12 2Δ P_L_-*ilvBN* Δ*ilvYC*::*kan*	K12 derivative with deletions of *ilvGM*, *ilvIH* and *ilvYC* genes, overexpression of *ilvBN* genes under control of “strong” constitutive promoter P_L_	This work
K12 *cat*-*ilvY*-P_*ilvC*_-*lacZ* 2Δ P_L_-*ilvBN* Δ*ilvYC*::*kan*	K12 2ΔP_L_-*ilvBN* Δ *ilvYC*::*kan* derivative with expression unit *cat*-*ilvY*-P_*ilvC*_ upstream of *lacZ* gene	This work
K12 2Δ P_L_-*ilvBN*^*fbr*^ Δ*ilvYC*::*kan*	K12 derivative with deletions of *ilvGM*, *ilvIH* and *ilvYC* genes, overexpression of *ilvBN*^*fbr*^ genes under control of “strong” constitutive promoter P_L_	This work
K12 2Δ P_L_-*ilvBN*^*fbr*^ ::*cat*-P_L_-*ilvBN*^*fbr*^	K12 derivative with deletions of *ilvGM* and *ilvIH* genes, overexpression of *ilvBN*^*fbr*^ genes under control of “strong” constitutive promoter P_L_(native locus) and insertion of additional copy of P_L_-*ilvBN*^*fbr*^ into *ppsA* locus	This work
K12 2Δ P_L_-*ilvBN*^*fbr*^ ::*cat*	K12 derivative with deletions of *ilvGM* and *ilvIH* genes, overexpression of *ilvBN*^*fbr*^ genes under control of “strong” constitutive promoter P_L_ and deletion of *ppsA* gene, marked with the *cat* gene	This work
K12 *cat*-*ilvY*-P_*ilvC*_-*lacZ* 2Δ P_L_-*ilvBN*^*fbr*^ Δ*ilvYC*::*kan*	K12 2Δ P_L_-*ilvBN*^*fbr*^ Δ*ilvYC*::*kan* derivative with expression unit *cat*-*ilvY*-P_*ilvC*_ upstream of *lacZ* gene	This work
K12 2Δ *cat*-*ilvY*-P_*ilvC*_-*ilvBN*^*fbr*^	K12 derivative with deletions of *ilvGM* and *ilvIH* genes, overexpression of *ilvBN*^*fbr*^ genes encoding feedback-resistant AHAS I under control of expression unit *cat*-*ilvY*-P_*ilvC*_	This work
K12 2Δ *ilvY*^*inactive*^-P_*ilvC*_-*ilvBN*^*fbr*^	K12 derivative with deletions of *ilvGM* and *ilvIH* genes, overexpression of *ilvBN*^*fbr*^ genes under control of expression unit *cat*-*ilvY*^*inactive*^-P_*ilvC*_ containing mutation in *ilvY* gene	This work
K12 Δ *ilvY* 2Δ *cat*-*ilvY*-P_*ilvC*_-*ilvBN*^*fbr*^	K12 derivative with deletions of *ilvGM*, *ilvIH* and *ilvY* (native locus) genes, overexpression of *ilvBN*^*fbr*^ genes under control of expression unit *cat*-*ilvY*-P_*ilvC*_	This work

^a^ VKPM, The Russian National Collection of Industrial Microorganisms

### Standard genetic engineering methods

Protocols for the genetic manipulation of *E*. *coli* and techniques for the isolation and manipulation of nucleic acids were described previously [35]. AccuTaq LA DNA polymerase (Sigma, USA) was used for PCR in accordance with the manufacturer’s instructions. All primers used in this work are listed in S1 Table. λRed-mediated integration was performed according to Datsenko and Wanner [37]. The recombinant plasmid pKD46 [37] with a temperature-sensitive replicon was used as the donor of the phage λ-derived genes responsible for the λRed-mediated recombination system.

### Construction of the *cat*-*ilvY*-P_*ilvC*_-*lacZ* expression cassette

To construct the *cat*-*ilvY*-P_*ilvC*_-*lacZ* expression unit, the *cat* gene was first introduced downstream of the *ilvY* gene on the chromosome of *E*. *coli* strain MG1655 using λRed-mediated integration. A DNA fragment bearing the λ*attL*-*cat*-λ*attR* cassette was amplified by PCR using the oligonucleotide primers P1 (for the *ilvY*-*attL* region) and P2 (for the *attR*-*ilvY* region), and the plasmid pMW118-λ*attL*-*cat*-λ*attR* [36] was used as the template. Second, the fragment *cat*-*ilvY*-P_*ilvC*_ including λ*attL*-*cat*-λ*attR*, the *ilvY* gene, and intergenic region *ilvY-ilvC* with the P_*ilvC*_ promoter, was PCR-amplified using the oligonucleotide primers P3 (for the *attL*-*lacZ* region) and P4 (for the *ilvCp*-*lacZ* region), and the chromosome of the *E*. *coli* strain MG1655 *cat*-*ilvY* was used as the template. The obtained PCR fragment was inserted into the *E*. *coli* MG1655/pKD46 chromosome region upstream of the *lacZ* gene by means of λRed-mediated integration. As a result, the strain *E*. *coli* MG1655 *cat*-*ilvY*-P_*ilvC*_-*lacZ* was obtained. The *cat-ilvY-*P_*ilvC*_*-lacZ* expression unit was transferred into several *E*. *coli* strains by P1 transduction [35].

### Construction of *E*. *coli* strains K12 2Δ and K12 3Δ

Deletion of the *ilvBN* operon was accomplished by means of λRed-mediated integration. A DNA fragment bearing the λ*attL*-*cat*-λ*attR* cassette was amplified by PCR using the primers ilvBN1 and ilvBN2, and the plasmid pMW118-λ*attL*-*cat*-λ*attR* was used as a template. The obtained 1713 bp PCR product was used for electroporation of the *E*. *coli* strain MG1655/pKD46. As a result, the *E*. *coli* MG1655 Δ*ilvBN* strain was obtained. Deletions of the *ilvIH* operon and *ilvGM* genes were constructed by the same approach used for the deletion of the *ilvBN* operon; the primers ilvIH1 and ilvIH2 were used for the *ilvIH* deletion, and primers ilvGM1 and ilvGM2 were used for the *ilvGM* deletion. The deletion of *ilvGM* genes was specially designed to minimize polarity effects on the expression of distal genes of the isoleucine-valine *ilvGMEDA* operon. A combination of Δ*ilvBN*, *ΔilvIH* and *ΔilvGM* deletions was accomplished by P1 transduction [35] with intermediate elimination of the chloramphenicol resistance marker. As a result, strains K12 2Δ (= K12 Δ*ilvIH* Δilv*GM*) and K12 3Δ (= K12 Δ*ilvIH* Δilv*GM* Δ*ilvBN*) were obtained. The strain K12 2Δ was prototrophic, therefore deletion of *ilvGM* genes did not prevent expression of distal genes of the isoleucine-valine operon.

### Construction of *E*. *coli* strains harboring different *ilvBN* and *ilvBN*^*fbr*^ expression units

The native regulatory region of the *ilvBN* operon was replaced with the phage lambda P_L_ promoter by λRed-mediated integration. For that purpose, we used the oligonucleotide ilvB-attR1, which is homologous to the region upstream of the *ilvB* gene and the region adjacent to the gene conferring antibiotic resistance, and the oligonucleotide ilvB-PLSD, which is homologous to both the *ilvB* region and the region downstream of the P_L_ promoter (for details of construction, see [38]). The strain K12 2Δ, with a single copy of the operon encoding AHAS I, was used for integrating the regulatory region *cat*-P_L_ upstream of the *ilvBN* operon. The obtained strain, K12 2Δ P_L_-*ilvBN*, was L-valine sensitive.

New L-valine-resistant spontaneous mutants of AHAS I were obtained from strain K12 2Δ P_L_-*ilvBN*. Spontaneous mutants that were resistant to L-valine were selected on plates with minimal medium that had been supplemented with 1 g/L L-valine. Strains that grew better on medium with 1 g/L L-valine were characterized. Among them, the enzyme containing a mutant small regulatory subunit IlvN^N17K^ demonstrated highest AHAS specific activity (for details of construction, see [38]). Thus, K12 2Δ P_L_-*ilvBN*^*fbr*^ was constructed.

The phage promoter P_L_ upstream of the *ilvBN*^*fbr*^ genes was substituted for the *cat*-*ilvY*-P_*ilvC*_ regulatory region using λRed-mediated integration, giving a strain with an autoinducible PFM for L-valine biosynthesis. To accomplish this aim, primers P7 and P8 were used.

To design an additional copy of the P_L_-*ilvBN*^*fbr*^ construct, a PCR fragment containing *cat*- P_L_-*ilvBN*^*fbr*^ was PCR-amplified by using primers ppsattRL and ppsilvN and integrated into *ppsA* locus of the chromosome of *E*. *coli* K12 3Δ by λRed-mediated integration. The resulting strain, K12 3Δ ::*cat*-P_L_-*ilvBN*^*fbr*^, was used as a donor for P1-transduction to combine two copies of P_L_-*ilvBN*^*fbr*^ at one chromosome yielding K12 2Δ P_L_-*ilvBN*^*fbr*^ ::*cat*-P_L_-*ilvBN*^*fbr*^. To demonstrate that deletion of the *ppsA* gene encoding phosphoenolpyruvate synthase, non-essential for L-valine biosynthesis, did not have a negative effect on the strain performance, the *ppsA* gene was inactivated in a fashion similar to the previously described method by using the primers ppsIL and ppsIR. The obtained strain, MG1655 Δ*ppsA*::*cat*, was used as donor for P1 transduction of the cassette Δ*ppsA*::*cat*.

### Construction of strains harboring *cat*-P_tac_-*lacZ*

The expression cassette *cat*-P_tac_-*lacZ*, kindly provided by Dr. Katashkina [36], contained the exporter gene *lacZ* under control of the hybrid promoter P_tac_, which contained the consensus sequences of the –35 and –10 regions from the natural promoters of the tryptophan and lactose (UV5) operon, respectively. This cassette was transferred into several strains by means of P1 transduction [35].

### Calculation of the translation initiation rate

The translation initiation rate, TIR, for the expression cassettes containing genes encoding feedback-resistant AHAS I under the control of different regulatory elements, i.e., P_L_-*ilvBN*^*fbr*^ and *ilvY*-P_*ilvC*_-*ilvBN*^*fbr*^ constructs, was calculated by using the Salis Lab RBS Calculator v 2.0 https://salislab.net/software/ [39].

### Construction of *ilvY*-deficient strains

An *ilvYC* deletion was constructed in two steps using λRed-mediated integration with oligonucleotide primers P5 and P6 and the plasmid pMW118-λ*attL*-*kan*-λ*attR* [36] as the template. As a result, the *E*. *coli* MG1655 Δ*ilvYC*::*kan* strain was obtained.

The inactivation of the *ilvY* gene in its native locus was done by introduction of microdeletion as follows. First, a PCR fragment harboring the λ*attL*-*cat*-λ*attR* cassette with the regions adjacent to an *ilvY* internal region was obtained using the oligonucleotide primers P9 and P10 and the plasmid pMIV5-JS as the template. The plasmid pMIV5-JS was constructed as described in [38]. As a result, the *E*. *coli* MG1655 Δ*ilvY*::*cat* strain, containing the chloramphenicol resistant marker (Cm^R^) in the *ilvY* coding region, was obtained. The cassette was transferred into strain K12 2Δ *cat*-*ilvY*-P_*ilvC*_-*ilvBN*^*fbr*^ by P1 transduction. After Cm^R^ marker elimination, the strain K12 2Δ Δ*ilvY cat*-*ilvY*-P_*ilvC*_-*ilvBN*^*fbr*^ was obtained.

Inactivation of the additional copy of the *ilvY* gene, a part of the *cat*-*ilvY*-P_*ilvC*_ cassette, was performed as follows. The *E*. *coli* K12 2Δ *cat*-*ilvY*-P_*ilvC*_-*ilvBN*^*fbr*^ strain was cured from the Cm^R^ marker by transient introduction of pMWts-λInt/Xis plasmid, which resulted in the markerless *E*. *coli* K12 2Δ *ilvY*-P_*ilvC*_-*ilvBN*^fbr^ strain. The *ilvYC* genes were deleted from the *E*. *coli* K12 2Δ *cat*-*ilvY*-P_*ilvC*_-*ilvBN*^*fbr*^ strain by P1 transduction as described above, using *E*. *coli* MG1655 Δ*ilvYC*::*kan* as a donor. Having obtained the λRed genes via the plasmid pKD46, the *E*. *coli* K12 2Δ *cat*-*ilvY*-P_*ilvC*_-*ilvBN*^*fbr*^ Δ*ilvYC*::*kan* strain was electrotransformed with the PCR fragment harboring the λ*attL*-*cat*-λ*attR* cassette with the regions adjacent to *ilvY* internal region. This PCR fragment was amplified with the oligonucleotide primers P11 and P12, and the chromosome of the *E*. *coli* MG1655 *ilvY*::*cat* strain was used as the template. As a result, the *E*. *coli* B7 K12 2Δ Δ*ilvYC*::*kan ilvY*::*cat*-P_*ilvC*_-*ilvBN*^*fbr*^ strain was obtained, which was then used as a donor strain to transduce the *ilvY*::*cat*-P_*ilvC*_- *ilvBN*^*fbr*^ cassette into the *E*. *coli* K12 2Δ *ilvY*-P_*ilvC*_- *ilvBN*^*fbr*^ strain. The P1 transduction was performed as described above. This process resulted in the strain *E*. *coli* K12 2Δ *ilvY*::*cat*-P_*ilvC*_- *ilvBN*^*fbr*^, which possesses only one active copy of the *ilvY* gene in its native locus due to inactivation of the *ilvY* gene copy in the *ilvY*::*cat*-P_*ilvC*_-*ilvBN*^*fbr*^ cassette as described above. The *cat* gene was eliminated using the transient introduction of the pMWts-λInt/Xis plasmid. As a result, the markerless *E*. *coli* B7 Δ*ilvGM* Δ*ilvIH ilvY*^inactive^-P_*ilvC*_-*ilvBN*4 strain was obtained.

### β-Galactosidase activity assay

Cells were grown to the mid-logarithmic phase in M9:LB (9:1, v/v) medium. The medium for strains having an *ilvYC* deletion and AHAS-deficient strains was additionally supplemented with Ile (25 mg/l) and Val (25 mg/l). The activity of β-galactosidase was measured according to Miller’s method [40]. The mean of triplicate experiments is presented; the standard deviation was less than 20%. MU = Miller’s units.

### AHAS activity assay

Cells were grown to the mid-logarithmic phase in M9: LB (9:1, v/v) medium. The activity of AHAS I in crude cell extracts was measured with or without the addition of 10 mM L-Val according to the assay described previously [41]. The mean of triplicate experiments is presented.

### Test tube fermentation conditions

Strains were each cultivated at 32°C for 18 hours in LB medium. Then, 0.2 mL of the obtained culture was inoculated into 2 mL of FM medium in 20 × 200 mm test-tubes and cultivated at 30°C for 60 hours on a rotary shaker at 250 rpm. After cultivation, the accumulated L-valine was measured using thin-layer chromatography (TLC). TLC plates (10 x 20 cm) were coated with 0.11 mm layers of Sorbfil silica gel containing nonfluorescent indicator (Sorbpolymer, Krasnodar, Russian Federation). Samples were applied to the plates with the Camag Linomat 5 sample applicator. The Sorbfil plates were developed with a mobile phase consisting of *iso*-propanol:ethylacetate:25% aqueous ammonia:water (16:16:5:10, v/v). A solution of ninhydrin (2%, w/v) in acetone was used as the visualizing reagent. After development, plates were dried and scanned with the Camag TLC Scanner 3 in absorbance mode with detection at 520 nm using winCATS software (version 1.4.2). Average data of 4 independent test tube fermentations are shown. Optical density at wavelength 540 nm, OD_540_, was measured by using Infinite M200 (Tecan, Austria).

## Results and discussion

### Properties of acetohydroxy acid-regulated expression unit based on the regulatory region of the *ilvC* gene

In the present work, the regulatory region of the *ilvC* gene was used as a metabolically regulated expression module in *E*. *coli*. Whereas the majority of isoleucine-valine biosynthetic genes, e.g., *ilvGMEDA* and *ilvBN* operons, are under negative control by the end products with participation of a transcription attenuation mechanism, the *ilvC* gene in *E*. *coli* is positively regulated by the intermediates of BCAA biosynthesis. 2-Acetolactate (AL) and 2-aceto-2-hydroxybutanoate (AHB), the products of the AHAS-mediated reactions and also the substrates for 2-acetohydroxy acid isomeroreductase (IlvC or KARI), are co-inducers of the transcription activator IlvY, which enhances *ilvC* gene expression [34]. Thus, IlvC synthesis is activated in the presence of its own substrates. Moreover, the *ilvC* gene has overlapping promoter regions with *ilvY* gene. Both genes are divergently transcribed in a coordinated fashion, and this coordination is achieved via supercoiling in the limited space between the two promoters [31].

To elucidate the ability of the promoter P_*ilvC*_ to be regulated metabolically, its functioning was studied in different genetic background that might alter the pools of inducer molecules. To this end, the transcriptional fusion expression cassette *cat*-*ilvY*-P_*ilvC*_-*lacZ* was constructed. The tested regulatory unit included the *ilvY* gene, encoding the LysR-type regulatory protein, and the intergenic region *ilvY-ilvC*, containing the P_*ilvC*_ promoter. The obtained expression cassette *cat*-*ilvY*-P_*ilvC*_-*lacZ* was transferred into the following strains, which differ in their ability to synthesize and metabolize AL (in this case, we focused on AL rather than AHB):

1. K12, wild-type strain with native AL synthesis and utilization;
2. K12 3Δ, AHAS-deficient strain with deletions of *ilvBN*, *ilvGM* and *ilvIH* genes and the absence of AL synthesis;
3. K12 2Δ P_L_-*ilvBN*, strain with increased AL synthesis due to the overexpression of *ilvBN* genes under the control of “strong” constitutive promoter P_L_; additionally, contains disruptions of Δ*ilvGM* and Δ*ilvIH* genes;
4. K12 2Δ P_L_-*ilvBN*^*fbr*^, strain similar to (iii), but instead of wild-type *ilvBN* genes, the mutant operon encoding feedback-resistant AHAS I, designated as *ilvBN*^*fbr*^, was used;
5. K12 2Δ P_L_-*ilvBN* Δ*ilvYC*::*kan*, strain with increased AL synthesis and defect in AL utilization due to the inactivation of *ilvYC* genes;
6. K12 2Δ P_L_-*ilvBN*^*fbr*^ Δ*ilvYC*::*kan*, strain similar to (v) but containing mutant *ilvBN*^*fbr*^ operon.

It should be noted that the deletion of *ilvGM* genes was specially constructed to minimize polarity effect on the expression of distal genes of the isoleucine-valine operon *ilvGMEDA*. A feedback-resistant AHAS I, containing the mutant small regulatory subunit IlvN^N17K^, was applied. This enzyme demonstrated more than 70% residual activity in the presence of 10 mM L-Val (see [38] for details).

As expected, specific β-galactosidase (LacZ) activity measurements in strains harboring the expression cassette *cat*-*ilvY*-P_*ilvC*_-*lacZ* indicated a correlation between the LacZ activity and the presumptive level of AL in a cell (Table 2). Thus, in the case of the AHAS-deficient strain K12 *cat*-*ilvY*-P_*ilvC*_-*lacZ* 3Δ, i.e., in the absence of co-inductor molecule synthesis, the activity was undetectable, while strain K12 *cat*-*ilvY*-P_*ilvC*_-*lacZ*, with native synthesis and utilization of AL, demonstrated the activity. Strain K12 *cat*-*ilvY*-P_*ilvC*_-*lacZ* 2Δ P_L_-*ilvBN*, possessing a relatively high level of AL synthesis, demonstrated LacZ activity comparable with that produced by the native LacZ regulation under isopropyl-β-D-thiogalactoside (IPTG) induction. Overexpression of the mutant operon *ilvBN*^*fbr*^, encoding feedback-resistant AHAS I, led to a further increase in LacZ activity, up to 4-fold higher than that of the strain harboring the wild-type AHAS I under the same expression conditions. The maximal expression from P_*ilvC*_ was provided by the *E*. *coli* strains modified to overexpress feedback-resistant AHAS I (the product of the *ilvBN*^*fbr*^ genes) and/or lacking the isomeroreductase KARI (IlvC) that normally metabolizes the inducer. In the case of KARI deficiency, there was no difference between LacZ activities in strains with wild-type and feedback-resistant AHAS I (Table 2).

**Table 2:** Activity of β-galactosidase LacZ in strains harboring the expression cassette *ilvY*-P_*ilvC*_-*lacZ* in various genetic backgrounds.

Strain	Description	LacZ activity, MU
K12 (IPTG, 1 mM)	Wild-type strain, native LacZ regulation under IPTG induction	1200
K12 *cat*-*ilvY*-P_*ilvC*_-*lacZ*	Wild-type strain harboring the expression cassette *cat*-*ilvY*-P_*ilvC*_-*lacZ*	150
K12 *cat*-*ilvY*-P_*ilvC*_-*lacZ* 3Δ	3Δ = Δ*ilvBN* Δ*ilvGM* Δ*ilvIH*, AL synthesis is blocked	≤10
K12 *cat*-*ilvY*-P_*ilvC*_-*lacZ* 2Δ P_L_-*ilvBN*	2Δ = Δ*ilvGM* Δ*ilvIH*, AL is synthesized by AHAS I	940
K12 *cat*-*ilvY*-P_*ilvC*_-*lacZ* 2Δ P_L_-*ilvBN*^*fbr*^	2Δ = Δ*ilvGM* Δ*ilvIH*, AL is synthesized by feedback-resistant AHAS I	3700
K12 *cat*-*ilvY*-P_*ilvC*_-*lacZ* 2Δ P_L_-*ilvBN* Δ*ilvYC*::*kan*	2Δ = Δ*ilvGM* Δ*ilvIH*, AL is synthesized by AHAS I + no utilization due to the inactivation of KARI	4400
K12 *cat*-*ilvY*-P_*ilvC*_-*lacZ* 2Δ P_L_-*ilvBN*^*fbr*^ Δ*ilvYC*::*kan*	2Δ = Δ*ilvGM* Δ*ilvIH*, AL is synthesized by feedback-resistant AHAS I + no utilization due to the inactivation of KARI	4400

The difference in LacZ activity between the tested strains can be caused, theoretically, not only by the difference in transcriptional level concerned with the AL inducer availability but also by other factors, such as significant differences in ribosomes, RNA polymerase pools, and mRNA stability, depending on the strain genotype. To confirm that the above factors had no essential impact, the same reporter, the *lacZ* gene, was put under the control of the constitutive promoter P_tac_ in the analyzed strains with different ability for AL synthesis. As the LacZ activity level was similar for all the above strains containing the same construct, *cat*-P_tac_-*lacZ* (S2 Table), the level of AL in the cells was likely the main reason for differences in LacZ activity between strains that possessed the expression cassette *cat*-*ilvY*-P_*ilvC*_-*lacZ* (Table 2).

Thus, the data show that P_*ilvC*_-dependent expression levels may vary over a broad range, by more than a factor of 400, because of dependence on the co-inducer pool, particularly AL.

### Application of positive feedback module for autoinducible production of L-valine by *E*. *coli*

**Fig 3:**
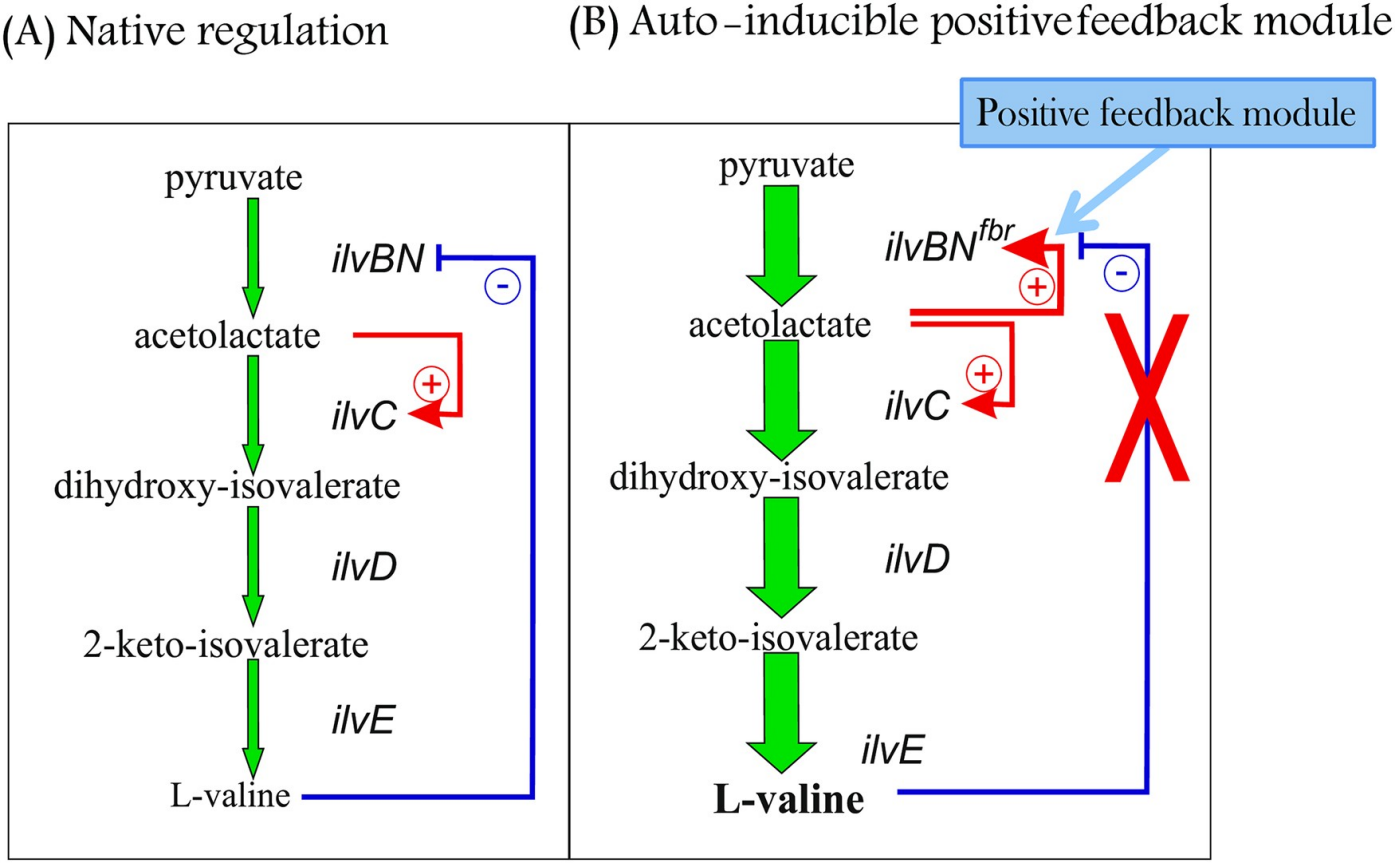
Schematics of L-valine biosynthesis. **a) Native regulation.** Negative feedback control: inhibition of AHAS I (IlvBN) activity by the pathway end product, L- valine. Positive control: AL induces its own utilization by KARI (IlvC). **b) Autoinducible positive feedback module.** Elimination of feedback control by protein modification (IlvBN^fbr^). Positive feedback loop: autoinducibility of AL synthesis due to the replacement of *ilvBN*^*fbr*^ regulatory region with an *ilvY*-P_*ilvC*_ unit. Accumulation of AL leads to the activation of transcription from P_*ilvC*_ and increased AHAS synthesis. Thus, AL activates its own synthesis and utilization.

In the present work, we developed an autoinducible gene expression module with a positive feedback loop, a so-called PFM. The idea is to place a biosynthetic gene under positive control of the product of a corresponding enzyme to incorporate a positive feedback unit into the biosynthetic pathway. The appearance of the product thus leads to activation of enzyme synthesis, which in turn results in the high accumulation of the product and a high yield of a final product. We realized this scheme by a model of an *E*. *coli* L-valine-producing strain (Fig 3).

As known, in *E*. *coli* K12 all the key enzymes of L-Val production are L-Val-sensitive: AHAS I (IlvBN) and AHAS III (IlvIH) [42]. AHAS II isozyme, L-Val-resistant, is inactive because of a frameshift mutation in *ilvG*. Thus, the wild-type strain *E*. *coli* K12 does not produce any detectable amounts of L-Val (Table 3); moreover, it does not grow in the presence of Val in the medium (minimal inhibiting concentration is less than 10 mg/L). As indicated above, a feedback-resistant AHAS I encoded by *ilvBN*^*fbr*^ operon was obtained and could be used for construction of L-Val-producing bacterium. It should be noted that in the wild-type *E*. *coli* cells both the operons, *ilvBN* and *ilvIH*, are subjected to transcription attenuation and their expression is depressed by BCAA [42]. So,the regulatory region of the *ilvBN*^*fbr*^ operon was replaced with the regulatory region of the *ilvC* gene. Thus, the product of the AHAS-mediated reaction, AL, activated the transcription from the P_*ilvC*_ promoter and increased AHAS I synthesis.

**Table 3:** Production of L-valine by modified *E*. *coli* strains harboring AHAS-encoding genes as a part of different expression units.

Strain	*ilvBN*^*fbr*^ allele	OD_540_	Production of L-Val, g/L
K12	no; only native AHAS	30.0	≤0.1
K12 2Δ P_L_-*ilvBN*^*fbr*^	1 copy; native locus; under control of ‘strong’ constitutive P_L_ promoter	20.7	4.3 ± 0.7
K12 2Δ P_L_-*ilvBN*^*fbr*^::*cat*-P_L_-*ilvBN*^*fbr*^	2 copies; native locus + Δ*ppsA* locus; both are under control of ‘strong’ constitutive P_L_ promoter	21.2	4.0 ± 0.4
K12 2Δ P_L_-*ilvBN*^*fbr*^ ::*cat*	1 copy; native locus; under control of ‘strong’ constitutive P_L_ promoter	21.6	4.4 ± 0.4
K12 2Δ *cat*-*ilvY*-P_*ilvC*_-*ilvBN*^*fbr*^	1 copy; native locus; under control of *ilvY*-P_*ilvC*_-based positive feedback regulatory element	21.6	6.1 ± 0.5

Since the native regulation of the *ilvBN* operon includes the attenuation of transcription by L-Val and L-Leu and seems to be not suitable for overproduction of L-Val, we decided to compare the performance of autoinducible expression modules based on the regulatory region of the *ilvC* gene with that of the”strong” constitutive promoter P_L_.

Both variants of transcriptional control, constitutive by the cassette P_L_-*ilvBN*^*fbr*^ or metabolic by the cassette *cat*-*ilvY*-P_*ilvC*_-*ilvBN*^*fbr*^, provided the similar level of AHAS I activity (Table 4). Nevertheless, the latter was preferable from the viewpoint of the final product accumulation, L-Val production was increased by more than 40% (Table 3). The enzyme activity level itself does not give enough information about transcription from corresponding promoters. Difference in enzyme activity can be caused by other reasons and particularly by the structure of the 5’-end of mRNA, which defines the ribosome binding. Nevertheless, for both expression cassettes, P_L_-*ilvBN*^*fbr*^ and *ilvY*-P_*ilvC*_-*ilvBN*^*fbr*^, the calculated TIRs were similar (458 for the former and 482 for the latter).

**Table 4:** Activity of AHAS I in *E*. *coli* strains harboring AHAS-encoding genes as a part of different expression units.

Strain	AHAS I activity, nmol/min*mg
K12 2Δ P_L_-*ilvBN*^*fbr*^	93 ± 43
K12 2Δ P_L_-*ilvBN*^*fbr*^ ::*cat*-P_L_-*ilvBN*^*fbr*^	83 ± 15
K12 2Δ P_L_-*ilvBN*^*fbr*^ ::*cat*	114 ± 12
K12 2Δ *cat*-*ilvY*-P_*ilvC*_-*ilvBN*^*fbr*^	90 ± 37

Notably, a further increase in *ilvBN*^*fbr*^ expression by the introduction of an additional copy of P_L_-*ilvBN*^*fbr*^ construct did not increase AHAS activity and L-valine production by K12 2Δ P_L_-*ilvBN*^*fbr*^ (Tables 3 and 4). Most likely, an excess of AHAS I accumulated in the insoluble fraction [43]. Therefore, metabolic control of *ilvBN*^*fbr*^ expression seems preferable to the ‘strong’ promoter control.

The K12 2Δ *cat*-*ilvY*-P_*ilvC*_-*ilvBN*^*fbr*^ strain contains two copies of *ilvY*: one in its native locus and one as a part of the *cat*-*ilvY*-P_*ilvC*_- *ilvBN*^*fbr*^ cassette. To exclude the possibility that the positive effect on L-Val production from the introduction of the cassette *cat*-*ilvY*-P_*ilvC*_-*ilvBN*^*fbr*^ was related to the amplification of positive regulator IlvY, we inactivated the additional copy of the *ilvY* gene that had been introduced upstream of the AHAS I genes. The copy in the upstream part of the *ilvBN*^*fbr*^ operon was precisely inactivated due to the introduction of a point mutation. A comparison of the resulting strain K12 2Δ *ilvY*^*inactive*^-P_*ilvC*_-*ilvBN*^*fbr*^ with the initial strain revealed that the inactivation of the additional copy of *ilvY* gene did not essentially influence L-Val accumulation (Table 5). Moreover, the precise elimination of the *ilvY* gene in its native locus also had no negative effect on L-Val production by K12 2Δ *cat*-*ilvY*-P_*ilvC*_-*ilvBN*^*fbr*^ (Table 5). Therefore, the usage of a P_*ilvC*_-based PFM for *ilvBN*^*fbr*^ operon expression, in addition to the amplification of the positive regulator *ilvY* gene, can be considered a reason for the strain improvement by the *cat*-*ilvY*-P_*ilvC*_-*ilvBN*^*fbr*^ cassette.

**Table 5:** Production of L-valine by modified *E*. *coli* strains harboring P_*ilvC*_-based PFM for *ilvBN*^*fbr*^ operon expression and different copy numbers of the positive regulator IlvY.

Strain	*ilvY* allele	OD_540_	Production of L-Val, g/L
K12 2Δ *cat*-*ilvY*-P_*ilvC*_-*ilvBN*^*fbr*^	2 copies; native locus + upstream *ilvBN*^*fbr*^ operon	21.5	5.7 ± 0.5
K12 2Δ *ilvY*^*inactive*^-P_*ilvC*_-*ilvBN*^*fbr*^	1 copy; native locus	22.7	5.7 ± 0.5
K12 2Δ Δ*ilvY cat*-*ilvY*-P_*ilvC*_-*ilvBN*^*fbr*^	1 copy; upstream *ilvBN*^*fbr*^ operon	20.5	5.5 ± 0.5

Strategies based on the usage of PFM are not limited to the IlvY\AL\P_*ilvC*_ autoinducible module. PFMs as described herein can be designed based on other LysR-type regulatory elements. For example, cysteine biosynthesis could be modified via an artificial PFM by using the O-acetyl-L-serine (OAS)/CysB-inducible promoters such as P_*cysP*_ and P_*cysK*_ for the autoinducible expression of genes encoding key enzyme(s) of cysteine biosynthesis (particularly, OAS biosynthesis), e.g., feedback-resistant serine acetyltransferese (CysE^fbr^). The regulatory factor CysB in complex with OAS activates the transcription of genes involved in high energy-consuming sulfate consumption and further reduction processes, which are undesirable in the absence of the OAS acceptor molecule [44–47]. Therefore, introduction of an artificial element containing the gene encoding CysE^fbr^ under the control of the OAS/CysB-activated promoter can lead to PFM formation and allow the use of CysB\OAS\P_*cysP*_ (or P_*cysK*,_ P_*cysD*,_ etc) as autoinducible regulatory elements. At the same time, since CysB regulon includes a wide range of genes, sometimes with unknown function, the application of OAS/CysB-based PFMs for industrial strain breeding seems a rather complicated task at this level.

Another PFM could be designed by using the regulatory region of the *metE* gene containing the *metR* gene, which encodes a LysR-type regulator, and the coupled divergent promoters P_*metR*_ and P_*metE*_. The P_*metE*_ promoter is regulated by the L-homocysteine/MetR complex [48]. Thus, such a kind of hypothetical PFM, MetR\L-homocysteine\P_*metE*_, can include the *metE* gene’s regulatory region to positively control the synthesis of L-homocysteine, an L-methionine precursor.

## Conclusions

In bacterial cells, many regulatory mechanisms are involved in negative feedback circuits that control the biosynthesis of metabolites, thereby preventing their excessive production, which is undesirable under certain conditions. Meanwhile, the activation of gene expression occurs in response to environmental or intracellular signals that indicate a necessity for adaptation to changing conditions (catabolism or transport of compounds, coordinated synthesis of separate structural elements in common biosynthetic pathways, stress defense, etc). In a native prokaryotic cell, examples of positive feedback circuits are rather rare and practically limited by signal transmission, such as “quorum sensing” [49–51]. In contrast, in artificial biological systems aimed to overproduce a target compound, such a strategy can be realized.

Here, we demonstrated an artificial way to regulate L-valine biosynthesis (Fig 3). As a first element of this artificial PFM, we used the modified AHAS I IlvBN^fbr^, which is an acetohydroxy acid synthase that is resistant to feedback inhibition. This element allows negative feedback control by the end product to be avoided. Replacement of the regulatory region of the *ilvBN*^*fbr*^ genes, which encode a modified AHAS I, with the regulatory region of the *ilvC* gene resulted in an artificial positive feedback loop. In this case, a portion of the AHAS I catalyzed the formation of reaction product, AL, which then simultaneously acted as a co-inducer with the regulator IlvY, induced transcription of the *ilvBN*^*fbr*^ operon and, thus further enhanced its own synthesis. At the same time, AL induced its own utilization by KARI according to the native regulatory mechanism. Thus, another portion of AL or AHB can be converted into an end product of the branched chain L-amino acid (L-valine, L-leucine or L-isoleucine) pathways.

The accumulation of AL, a product of the AHAS-mediated reaction, will thus lead to the activation of transcription from P_*ilvC*_ and increased AHAS synthesis. Therefore, AHAS synthesis is activated by its own product. Such an autoinducible PFM can supply an appropriate level of AHAS and the consequent AL, the precursor of the final product, L-valine. Introduction of such a PFM leads to L-Val overaccumulation and may have a practical impact.

The described strategy based on the usage of PFM is not limited to BCAA biosynthesis and could be applied for the breeding of industrial strains producing other essential metabolites, which broadens the set of metabolic engineering tools.

## Supporting information

S1 Table Sequences of the PCR primers used in this study. (DOCX)

S2 Table Activity of β-galactosidase LacZ in strains harboring the expression cassette *cat*-P_tac_-*lacZ* in various genetic background. (DOCX)

## References

[1] Singh R, Kumar M, Mittal A, Mehta PK. Microbial metabolites in nutrition, healthcare and agriculture. 3 Biotech. Springer; 2017; 7(1):15. 10.1007/s13205-016-0586-4 PMC5385174 28391479

[2] Oldiges M, Eikmanns BJ, Blombach B. Application of metabolic engineering for the biotechnological production of L-valine. Applied microbiology and biotechnology. Springer; 2014; 98(13):5859–70. 10.1007/s00253-014-5782-8 24816722

[3] Woolston BM, Edgar S, Stephanopoulos G. Metabolic engineering: past and future. Annual review of chemical and biomolecular engineering. Annual Reviews; 2013; 4:259–88. 10.1146/annurev-chembioeng-061312-103312 23540289

[4] Becker J, Wittmann C. Systems and synthetic metabolic engineering for amino acid production—the heartbeat of industrial strain development. Current opinion in biotechnology. Elsevier; 2012; 23(5):718–26. 10.1016/j.copbio.2011.12.025 22244788

[5] Ikeda M. Amino acid production processes. Microbial production of L-amino acids. Springer; 2003; 79:1–35. 10.1007/3-540-45989-8_1 12523387

[6] Bradley RW, Buck M, Wang B. Tools and principles for microbial gene circuit engineering. Journal of molecular biology. Elsevier; 2016; 428(5):862–88. 10.1016/j.jmb.2015.10.004 26463592

[7] Xiang Y, Dalchau N, Wang B. Scaling up genetic circuit design for cellular computing: advances and prospects. Natural computing. Springer; 2018; 17(4):833–53. 10.1007/s11047-018-9715-9 PMC6244767 30524216

[8] Pinto D, Vecchione S, Wu H, Mauri M, Mascher T, Fritz G. Engineering orthogonal synthetic timer circuits based on extracytoplasmic function sigma factors. Nucleic acids research. Oxford University Press; 2018; 46(14):7450–64. 10.1093/nar/gky614 PMC6101570 29986061

[9] Liu Q, Schumacher J, Wan X, Lou C, Wang B. Orthogonality and burdens of heterologous and gate gene circuits in *E*. *coli*. ACS synthetic biology. ACS Publications; 2018; 7(2):553–64. 10.1021/acssynbio.7b00328 PMC5820654 29240998

[10] Blazeck J, Alper HS. Promoter engineering: recent advances in controlling transcription at the most fundamental level. Biotechnol J. 2013; 8(1):46–58. 10.1002/biot.201200120 22890821

[11] Nijland R, Lindner C, Van Hartskamp M, Hamoen LW, Kuipers OP. Heterologous production and secretion of *Clostridium perfringens* beta-toxoid in closely related Gram-positive hosts. Journal of biotechnology. Elsevier; 2007; 127(3):361–72. 10.1016/j.jbiotec.2006.07.014 16959352

[12] Kerovuo J, Von Weymarn N, Povelainen M, Auer S, Miasnikov A. A new efficient expression system for *Bacillus* and its application to production of recombinant phytase. Biotechnology letters. Springer; 2000; 22(16):1311–7.

[13] Singh V. Recent advancements in synthetic biology: current status and challenges. Gene. 2014; 535(1):1–11 10.1016/j.gene.2013.11.025 24269673

[14] Esvelt KM, Wang HH. Genome-scale engineering for systems and synthetic biology. Molecular systems biology. EMBO Press; 2013; 9(1):641. 10.1038/msb.2012.66 PMC3564264 23340847

[15] Zhang F, Carothers JM, Keasling JD. Design of a dynamic sensor-regulator system for production of chemicals and fuels derived from fatty acids. Nat Biotechnol. 2012; 30(4):354–9. 10.1038/nbt.2149 22446695

[16] Farmer WR, Liao JC. Improving lycopene production in *Escherichia coli* by engineering metabolic control. Nature biotechnology. Nature Publishing Group; 2000; 18(5):533. 10.1038/75398 10802621

[17] Wang X, Zhang H, Quinn PJ. Production of L-valine from metabolically engineered *Corynebacterium glutamicum*. Applied microbiology and biotechnology. Springer; 2018; 102(10):4319–30. 10.1007/s00253-018-8952-2 29594358

[18] Park JH, Kim TY, Lee KH, Lee SY. Fed-batch culture of *Escherichia coli* for L-valine production based on *in silico* flux response analysis. Biotechnology and bioengineering. Wiley Online Library; 2011; 108(4):934–46. 10.1002/bit.22995 21404266

[19] Blombach B, Schreiner ME, Bartek T, Oldiges M, Eikmanns BJ. *Corynebacterium glutamicum* tailored for high-yield L-valine production. Applied microbiology and biotechnology. Springer; 2008; 79(3):471–9. 10.1007/s00253-008-1444-z 18379776

[20] Park JH, Lee KH, Kim TY, Lee SY. Metabolic engineering of *Escherichia coli* for the production of L-valine based on transcriptome analysis and *in silico* gene knockout simulation. Proceedings of the National Academy of Sciences. National Acad Sciences; 2007; 104(19):7797–802. 10.1073/pnas.0702609104 PMC1857225 17463081

[21] Parsek MR, McFall SM, Shinabarger DL, Chakrabarty A. Interaction of two LysR-type regulatory proteins CatR and ClcR with heterologous promoters: functional and evolutionary implications. Proceedings of the National Academy of Sciences. National Acad Sciences; 1994; 91(26):12393–7. 10.1073/pnas.91.26.12393 PMC45444 7809047

[22] Schell MA. Molecular biology of the LysR family of transcriptional regulators. Annual Reviews in Microbiology. Annual Reviews 4139 El Camino Way, PO Box 10139, Palo Alto, CA 94303–0139, USA; 1993; 47(1):597–626. 10.1146/annurev.mi.47.100193.003121 8257110

[23] Lindquist S, Lindberg F, Normark S. Binding of the *Citrobacter freundii* AmpR regulator to a single DNA site provides both autoregulation and activation of the inducible *ampC* beta-lactamase gene. Journal of bacteriology. Am Soc Microbiol; 1989; 171(7):3746–53. 10.1128/jb.171.7.3746-3753.1989 PMC210120 2786868

[24] Maddocks SE, Oyston PC. Structure and function of the LysR-type transcriptional regulator (LTTR) family proteins. Microbiology. Microbiology Society; 2008; 154(12):3609–23. 10.1099/mic.0.2008/022772-0 19047729

[25] Knapp GS, Hu JC. Specificity of the *E*. *coli* LysR-type transcriptional regulators. PloS one. Public Library of Science; 2010; 5(12):e15189. 10.1371/journal.pone.0015189 PMC3004787 21187915

[26] Jovanovic M, Lilic M, Savic DJ, Jovanovic G. The LysR-type transcriptional regulator CysB controls the repression of *hslJ* transcription in *Escherichia coli*. Microbiology (Reading, Engl). 2003; 149(Pt 12):3449–59. 10.1099/mic.0.26609-0 14663078

[27] Weissbach H, Brot N. Regulation of methionine synthesis in *Escherichia coli*. Molecular microbiology. Wiley Online Library; 1991; 5(7):1593–7. 10.1111/j.1365-2958.1991.tb01905.x 1943695

[28] Membrillo-Hernández J, Coopamah MD, Channa A, Hughes MN, Poole RK. A novel mechanism for upregulation of the *Escherichia coli* K-12 *hmp* (flavohaemoglobin) gene by the “NO releaser”, S-nitrosoglutathione: nitrosation of homocysteine and modulation of MetR binding to the *glyA-hmp* intergenic region. Molecular microbiology. Wiley Online Library; 1998; 29(4):1101–12. 10.1046/j.1365-2958.1998.01000.x 9767577

[29] Caldara M, Charlier D, Cunin R. The arginine regulon of *Escherichia coli*: whole-system transcriptome analysis discovers new genes and provides an integrated view of arginine regulation. Microbiology. Microbiology Society; 2006; 152(11):3343–54. 10.1099/mic.0.29088-0 17074904

[30] Kiupakis AK, Reitzer L. ArgR-independent induction and ArgR-dependent superinduction of the *astCADBE* operon in *Escherichia coli*. Journal of bacteriology. Am Soc Microbiol; 2002; 184(11):2940–50. 10.1128/JB.184.11.2940-2950.2002 PMC135064 12003934

[31] Opel ML, Hatfield GW. DNA supercoiling-dependent transcriptional coupling between the divergently transcribed promoters of the *ilvYC* operon of *Escherichia coli* is proportional to promoter strengths and transcript lengths. Molecular microbiology. Wiley Online Library; 2001; 39(1):191–8. 10.1046/j.1365-2958.2001.02249.x 11123701

[32] Rhee KY, Opel M, Ito E, Hung S, Arfin SM, Hatfield GW. Transcriptional coupling between the divergent promoters of a prototypic LysR-type regulatory system, the *ilvYC* operon of *Escherichia coli*. Proceedings of the National Academy of Sciences. National Acad Sciences; 1999; 96(25):14294–9. 10.1073/pnas.96.25.14294 PMC24430 10588699

[33] Rhee KY, Senear DF, Hatfield GW. Activation of gene expression by a ligand-induced conformational change of a protein-DNA complex. Journal of Biological Chemistry. ASBMB; 1998; 273(18):11257–66. 10.1074/jbc.273.18.11257 9556617

[34] Wek RC, Hatfield GW. Transcriptional activation at adjacent operators in the divergent-overlapping *ilvY* and *ilvC* promoters of *Escherichia coli*. J Mol Biol. 1988; 203(3):643–63. 3062177 10.1016/0022-2836(88)90199-4

[35] Sambrook J, Fritsch EF, Maniatis T, others. Molecular cloning: a laboratory manual. Cold spring harbor laboratory press; 1989.

[36] Katashkina J, Skorokhodova AY, Zimenkov D, Gulevich AY, Minaeva N, Doroshenko V, et al Tuning the expression level of a gene located on a bacterial chromosome. Molecular Biology. Springer; 2005; 39(5):719–26. 16240716

[37] Datsenko KA, Wanner BL. One-step inactivation of chromosomal genes in *Escherichia coli* K-12 using PCR products. Proc Natl Acad Sci USA. 2000; 97(12):6640–5. 10.1073/pnas.120163297 10829079 PMC18686

[38] Sycheva EV, Serebryanyy VA, Yampolskaya TA, Preobrazhenskaya ES, Stoynova NV. Mutant acetolactate synthase and a method for producing branched-chain L-amino acids. US Patent 9,279,137; 2016;

[39] Salis HM, Mirsky EA, Voigt CA. Automated design of synthetic ribosome binding sites to control protein expression. Nature biotechnology. Nature Publishing Group; 2009; 27(10):946. 10.1038/nbt.1568 PMC2782888 19801975

[40] Miller J. Experiments in molecular genetics. Cold Spring Laboratory Press Cold Spring Harbor, NY; 1972;

[41] Bauerle R, Freundlich M, Størmer F, Umbarger H. Control of isoleucine, valine and leucine biosynthesis: II. Endoproduct inhibition by valine of acetohydroxy acid synthetase in *Salmonella typhimurium*. Biochimica et Biophysica Acta (BBA)-Specialized Section on Enzymological Subjects. Elsevier; 1964; 92(1):142–9. 14243762

[42] Umbarger H. Biosynthesis of the branched-chain amino acids. *Escherichia coli* and *Salmonella typhimurium*: cellular and molecular biology. American Society for Microbiology; 1987; 352–67.

[43] Hill MC, Pang SS, Duggleby GR. Purification of *Escherichia coli* acetohydroxyacid synthase isoenzyme II and reconstitution of active enzyme from its individual pure subunits. Biochemical Journal. Portland Press Limited; 1997; 327(3):891–8. 10.1042/bj3270891 PMC1218872 9581571

[44] Lochowska A, Iwanicka-Nowicka R, Zaim J, Witkowska-Zimny M, Bolewska K, Hryniewicz MM. Identification of activating region (AR) of *Escherichia coli* LysR-type transcription factor CysB and CysB contact site on RNA polymerase alpha subunit at the *cysP* promoter. Molecular microbiology. Wiley Online Library; 2004; 53(3):791–806. 10.1111/j.1365-2958.2004.04161.x 15255893

[45] Van der Ploeg JR, Eichhorn E, Leisinger T. Sulfonate-sulfur metabolism and its regulation in *Escherichia coli*. Arch Microbiol. 2001; 176(1–2):1–8. 11479697 10.1007/s002030100298

[46] Kredich NM. The molecular basis for positive regulation of *cys* promoters in *Salmonella typhimurium* and *Escherichia coli*. Mol Microbiol. 1992; 6(19):2747–53. 1435253 10.1111/j.1365-2958.1992.tb01453.x

[47] Monroe R, Ostrowski J, Hryniewicz M, Kredich N. *In vitro* interactions of CysB protein with the *cysK* and *cysJIH* promoter regions of *Salmonella typhimurium*. Journal of bacteriology. Am Soc Microbiol; 1990; 172(12):6919–29. 10.1128/jb.172.12.6919-6929.1990 PMC210811 2254265

[48] Cai X-Y, Maxon ME, Redfield B, Glass R, Brot N, Weissbach H. Methionine synthesis in *Escherichia coli*: effect of the MetR protein on *metE* and *metH* expression. Proceedings of the National Academy of Sciences. National Acad Sciences; 1989; 86(12):4407–11. 10.1073/pnas.86.12.4407 PMC287278 2543976

[49] Poellinger KA, Lee JP, Parales JV, Greenberg EP. Intragenic suppression of a *luxR* mutation: characterization of an autoinducer-independent LuxR. FEMS Microbiol Lett. 1995; 129(1):97–101. 10.1016/0378-1097(95)00145-U 7781994

[50] Sayut DJ, Kambam PKR, Sun L. Noise and kinetics of LuxR positive feedback loops. Biochem Biophys Res Commun. 2007; 363(3):667–73. 10.1016/j.bbrc.2007.09.057 17905197

[51] Bansal K, Yang K, Nistala GJ, Gennis RB, Bhalerao KD. A positive feedback-based gene circuit to increase the production of a membrane protein. J Biol Eng. 2010; 4:6 10.1186/1754-1611-4-6 20500847 PMC2885990

