# Supplementary material for: Engineering Escherichia coli for autoinducible production of L-valine: An example of an artificial positive feedback loop in amino acid biosynthesis
Source: PLoS One. 2019 Apr 25;14(4):e0215777. doi: 10.1371/journal.pone.0215777 (PMC6483228; doi:10.1371/journal.pone.0215777)
Supplement: S1 Table — (DOCX) [file pone.0215777.s001.docx]

**Table S1**. **Sequences of the PCR primers used in this study.**

| **No.** | **Sequence (5′ → 3′)** | **Description** |
| --- | --- | --- |
| P1 | gagctgggctacgattgccacgacgaaaccaataactgaagcctgcttttttatactaagttgg | Integration of the *cat* gene downstream of the *ilvY* gene |
| P2 | ttttccctaacccgccaaaaagaacctgaacgccggcgctcaagttagtataaaaaagctgaac | Integration of the *cat* gene downstream of the *ilvY* gene |
| P3 | ttgtgagcggataacaatttcacacaggaaacagcttgaagcctgctttt ttatactaagttgg | Integration of the *cat*-*ilvY*-P_ilvC_ expression unit upstream of the lacZ gene |
| P4 | taaaacgacggccagtgaatccgtaatcatggtcatggtgattcctcgtg atgttgtgcttctt | Integration of the *cat*-*ilvY*-P_ilvC_ expression unit upstream of the lacZ gene |
| ilvBN1 | taaacatcgtcggatcggactgattacgctgcactttgaagcctgctttttatactaag  ttgg | Deletion of *ilvBN* operon |
| ilvBN2 | tcccggaaagtcggcccagaagaaaaggactggagccgctcaagttagtataaaaaagct  gaac | Deletion of *ilvBN* operon |
| ilvIH1 | ttcacctttcctcctgtttattcttattacccctgaagcctgcttttttatactaagttgg | Deletion of *ilvIH* operon |
| ilvIH2 | acatgttgggctgtaaattgcgcattgagatcattccgctcaagttagtataaaaaagcgaac | Deletion of *ilvIH* operon |
| ilvGM1 | tttctcaagattcaggacggggaactaactatgaatgaagcctgcttttttatactaagttgg | Deletion of *ilvGM* genes |
| ilvGM2 | tcagctttcttcgtggtcatttttatattccttttgcgctcaagttagtataaaaaagctgaac | Deletion of *ilvGM* genes |
| ilvB-attR1 | ccgcaggcgactgacgaaacctcgctccggcggggtcgctcaagttagtataaaaaagctgaac | Replacement of the native regulatory region of the *ilvBN* operon with phage lambda P_L_ promoter |
| ilvB-PLSD | tgcccgaacttgccatgctccagtctccttcttctgagct gtttccttctagacggccaatgct | Replacement of the native regulatory region of the *ilvBN* operon with phage lambda P_L_ promoter |
| P5 | gaccggcccgctgcacacccagttcggatatcacatcgctcaagttagtataaaaaagctgaac | The *ilvYC* deletion |
| P6 | gctgggctacgattgccacgacgaaaccaataaccctgaagcctgcttttttatactaagttgg | The *ilvYC* deletion |
| P7 | atgttgtgcccgaacttgccatgctccagtctccttggtg attcctcgtgatgttgtgct  tctt | Replacement of phage promoter P_L_ upstream of the *ilvBN^fbr^* operon with *cat*-*ilvY*-P_ilvC_ regulatory region |
| P8 | ccgcaggcgactgacgaaacctcgctccggcggggttgaagcctgcttttttatactaag  ttgg | Replacement of phage promoter P_L_ upstream of the *ilvBN^fbr^* operon with *cat*-*ilvY*-P_ilvC_ regulatory region |
| P9 | acgtcaacatcgagggctgtccctgtggatttacgctgaagcctgcttttttatactaag  ttgg | The *ilvY* deletion in native locus |
| P10 | ggcggctttccgccagatgcaggaaggttttcagatcgctcaagttagtataaaaaagctgaac | The *ilvY* deletion in native locus |
| P11 | gctgaatctgccgtgaga | The *ilvY* deletion from the *cat*-*ilvY*-P_ilvC_-*ilvBN^fbr^* cassette |
| P12 | ctggcgcagattcagtgt | The *ilvY* deletion from the *cat*-*ilvY*-  P_ilvC_-*ilvBN^fbr^* cassette |
| ppsattRL | tgtccaacaatggctcgtaaccgctggtgctttggtcgctcaagttagtataaaaaagctgaac | Construction of the cassette ∆*ppsA*::*cat*-P_L_-*ilvBN^fbr^* |
| ppsilvN | ttatttcttcagttcagccaggcttaaccaggtttgttactgaaaaaacaccgcgatcttgtt | Construction of the cassette ∆*ppsA*::*cat*-P_L_-*ilvBN^fbr^* |
| ppsIL | accccggcgactaaacgccgccggggatttattttatgaagcctgcttttttatactaag | Deletion of *ppsA* gene |
| ppsIR | tcaaaccgttcatttatcacaaaaggattgttcgatcgctcaagttagtataaaaaagct | Deletion of *ppsA* gene |
