# Supplementary material for: Engineering Escherichia coli for autoinducible production of L-valine: An example of an artificial positive feedback loop in amino acid biosynthesis
Source: PLoS One. 2019 Apr 25;14(4):e0215777. doi: 10.1371/journal.pone.0215777 (PMC6483228; doi:10.1371/journal.pone.0215777)
Supplement: S2 Table — (DOCX) [file pone.0215777.s002.docx]

**Table S2.** **Activity of β-galactosidase LacZ in strains harboring the expression cassette *cat*-P_tac_-*lacZ* in various genetic backgrounds**

| **Strain** | **LacZ activity, MU** |
| --- | --- |
| K12 *cat*-P_tac_-*lacZ* | 14000 |
| K12 *cat*-P_tac_-*lacZ* 3Δ | 15000 |
| K12 *cat*-P_tac_-*lacZ* 2Δ P_L_-*ilvBN* | 15000 |
| K12 *cat*-P_tac_-*lacZ* 2Δ P_L_-*ilvBN^fbr^* | 16000 |
